# Supplementary material for: Determinants of Stunting Among Children Aged 0.5 to 12 Years in Peninsular Malaysia: Findings from the SEANUTS II Study
Source: Nutrients. 2025 Jul 17;17(14):2348. doi: 10.3390/nu17142348 (PMC12299504; doi:10.3390/nu17142348)
Supplement: Supplementary file 1 [file nutrients-17-02348-s001.zip › nutrients-3700671-supplementary.pdf]

Supplementary Tables

Table S1 Univariate Odds Ratio for Child and Maternal Characteristics Related to Stunting.

Parameter	OR	95%CI	p-value
**Child Characteristics**			
**Age group (Years)**			
Infants (0.5-0.9 years)	3.20	1.41-7.26	0.005
Toddlers (1.0-3.9 years)	2.82	1.89-4.21	<0.001
Preschoolers (4.0-6.9 years)	1.63	1.07-2.49	0.023
School-aged children (7.0-12.9 years)	1.00		
**Sex**			
Girls	1.11	0.80-1.53	0.552
Boys	1.00		
**Residential areas**			
Rural	1.34	0.96-1.88	0.086
Urban	1.00		
**Ethnicity**			
Chinese	0.44	0.30-0.65	<0.001
Indian	0.19	0.07-0.50	0.001
Others	0.49	0.09-2.61	0.400
Malay	1.00		
**Birth Weight (n=2660)**			
Low (<2.5 kg)	2.37	1.47-3.82	<0.001
Normal (2.5 kg and above)	1.00		
**Household size (n=2964)**			
5 or more people	1.50	1.05-2.14	0.026
<5 people	1.00		
**Number of Siblings (n=2945)**			
1-2 siblings	1.69	1.16-2.45	0.006
3 or more siblings	1.73	1.10-2.73	0.019
No siblings	1.00		
**Parent Characteristics**			
**Maternal Age Group (n=2927)**			
< 30.0 years	1.63	0.87-3.06	0.130
30.0-39.9 years	2.06	1.40-3.03	<0.001
> 39.9 years	1.00		

**Maternal Height (n=2876)**			
<150 cm	2.60	1.67-4.07	<0.001
≥ 150 cm	1.00		
**Maternal Employment Status (n=2933)**			
Not Working	1.15	0.82-1.61	0.421
Working	1.00		
**Maternal Educational level (n=2943)**			
Non-schooling/primary school	1.07	0.50-2.27	0.859
Secondary school	0.93	0.66-1.30	0.671
Tertiary school (College/University)	1.00		
**Total Monthly Household Income (n=2916)**			
B40 (≤MYR 4,850)	1.62	0.77-3.38	0.201
M40 (MYR 4,851 – 10,959)	1.64	0.76-3.55	0.208
T20 (≥MYR 10,959)	1.00		
**Monthly Household Food Expenditure (n=2895)**			
< MYR 783	1.27	0.91-1.77	0.157
≥ MYR 783	1.00		
**Parental Smoking (n=2896)**			
No	0.75	0.54-1.05	0.090
Yes	1.00		

Table S2 Univariate Odds Ratio for Sanitation and Hygiene Practices of the Children and Mothers.

Parameter	OR	95%CI	p-value
**Sanitation Factor**			
**Improved Sanitation Facilities (n=2970)**			
No	1.04	0.59-1.83	0.906
Yes	1.00		
**Children's Hygiene Practices**			
**Washing hands before meals and after using the toilets (n=2935)**			
No	1.20	0.52-2.77	0.668
Yes	1.00		
**Washing hands using soap (n=2901)**			
No	1.36	0.81-2.27	0.241
Yes	1.00		
**Wearing shoes outside the house (n=2950)**			
No	0.89	0.57-1.38	0.595
Yes	1.00		
**Mothers' handwashing practices**			
**Before preparing food (n=2969)**			
No	1.10	0.60-1.99	0.761
Yes	1.00		
**After using the toilets (n=2967)**			
No	0.88	0.40-1.95	0.759
Yes	1.00		
